# Obesity, cytokines and psychopathology in patients with chronic schizophrenia

**DOI:** 10.3389/fpsyt.2025.1574041

**Published:** 2025-07-28

**Authors:** Menghan Lv, Xuan Wang, Xiayue He, Zhiren Wang, Xiaohong Li, Yunlong Tan, Xiang Yang Zhang

**Affiliations:** ^1^ Peking University HuiLongGuan, Clinical Medical School, Beijing Huilongguan Hospital, Beijing, China; ^2^ Hefei Fourth People’s Hospital, Anhui Mental Health Center, Affiliated Psychological Hospital of Anhui Medical University, Hefei, China

**Keywords:** schizophrenia, cytokine, obesity, lipid profiles, PANSS

## Abstract

**Background:**

Obesity and dysregulated cytokine levels are prevalent in schizophrenia patients undergoing antipsychotic treatment. While cytokines are implicated in obesity, their relationship with psychopathology in schizophrenia remains underexplored. This study investigated associations between body mass index (BMI), cytokine levels, and clinical symptoms in chronic schizophrenia patients.

**Methods:**

In this cross-sectional study,201chronic schizophrenia patients (Chinese Han population) were stratified into high BMI (BMI≥25kg/m^2)^ and low BMI (BMI<25kg/m^2^) groups. Psychopathology was assessed using the Positive and negative Syndrome Scale (PANSS). Serum cytokine (IL-2, IL-6, TNF-α) and metabolic parameters were measured in 69 participants.

**Results:**

A significant negative correlation was observed between BMI and IL-2(*p*=0.013). TNF-α levels inversely correlated with PANSS total (*p*=0.010) and general psychopathology scores(*p*=0.042). The high BMI group exhibited lower PANSS negative subscores and elevated glucose, triglycerides (TG) and apolipoprotein B (ApoB) compared to the low BMI group(all *p*<0.05). Multivariate regression identified IL-2 as an independent factor associated with lower BMI, while TNF-α independently contributed to general psychopathology.

**Conclusions:**

Higher BMI in chronic schizophrenia is associated with reduced IL-2 levels, attenuated negative symptoms, and adverse lipid profiles. TNF-α may modulate psychopathology severity. These findings highlight complex interactions between metabolic dysregulation, immune markers, and clinical manifestations in schizophrenia.

## Introduction

The global burden of obesity has escalated dramatically in recent decades, emerging as a critical public health challenge across diverse populations (1). Individuals diagnosed with schizophrenia are disproportionately affected by this metabolic epidemic, with prevalence rates of overweight and obesity exceeding 40-60%, nearly double that of the general population (2–5). This disparity is largely attributable to the metabolic adverse effects of antipsychotic medications, particularly second-generation agents which disrupt energy homeostasis through histaminergic, serotonergic and dopaminergic receptor antagonism, leading to hyperphagia, insulin resistance, and dyslipidemia (6–8). The consequences are dire: obesity in schizophrenia contributes to a 20-year reduction in life expectancy, primarily driven by cardiovascular disease, diabetes, and metabolic syndrome, while also exacerbating treatment nonadherence and relapse rates (9–11). These intertwined metabolic and psychiatric challenges underscore the urgent need to elucidate the biological mechanisms linking obesity to schizophrenia progression.

Concurrently, chronic low-grade inflammation has emerged as a shared pathophysiological hallmark of both obesity and schizophrenia (12, 13). In obesity, hypertrophic adipose tissue secretes proinflammatory cytokines, including interleukin-6 (IL-6), tumor necrosis factor-alpha (TNF-α), and monocyte chemoattractant protein-1 (MCP-1), which perpetuate systemic insulin resistance and endothelial dysfunction via NF-κB and JNK signaling pathways (14–16). In schizophrenia, meta-analyses consistently report elevated levels of inflammatory markers such as C-reactive protein (CRP), soluble IL-2 receptor (sIL-2R), and IL-6, suggesting immune dysregulation may contribute to neuro-progressive processes, including synaptic pruning deficits, microglial activation, and dopaminergic instability (17–19). Notably, genetic polymorphisms in cytokine genes have been linked to antipsychotic-induced weight gain, further implicating immune-metabolic crosstalk in this population (20–22). Despite these overlaps, the bidirectional relationship between obesity-associated inflammation and schizophrenia symptomatology remains poorly characterized, particularly in non-Western cohorts where genetic, dietary, and environmental factors may uniquely modulate immune responses.

A critical gap persists in understanding how specific cytokines correlate with psychopathological dimensions across body mass index (BMI) strata in schizophrenia. While studies in general populations demonstrate robust associations between obesity, elevated IL-6, and depressive symptoms, findings in schizophrenia are inconsistent (23, 24). For instance, some reports suggest TNF-α and IL-6 elevations correlate with cognitive deficits and negative symptoms (25), whereas others observe no significant relationships, likely due to heterogeneous study designs, unmeasured confounders, or insufficient adjustment for metabolic comorbidities (26, 27). Furthermore, the role of IL-2 a cytokine pivotal for T-cell regulation and immune tolerance—remains underexplored in schizophrenia-related obesity, despite evidence linking IL-2 deficiency to impaired lipid metabolism and adipocyte dysfunction in preclinical models (28, 29).

To address these gaps, this cross-sectional study investigated the interplay between BMI, cytokine profiles, and psychopathology in a well-characterized cohort of chronic schizophrenia patients from the Han Chinese population. By integrating metabolic, immunological, and psychiatric assessments, this study advances our understanding of the immune-metabolic axis in schizophrenia, offering potential biomarkers for obesity risk and novel targets for adjunctive therapies to mitigate both psychiatric and cardiometabolic morbidity.

## Methods

This study enrolled 201 patients diagnosed with chronic schizophrenia from Beijing Hui-Long-Guan Hospital, a municipal psychiatric facility in Beijing, China. Participants were recruited based on the following inclusion criteria:(1) age 35–65 years, Han Chinese; (2) diagnosis of schizophrenia based on DSM-V criteria by 2 experienced psychiatrists; (3) at least 5 years of illness course; (4) stable doses of antipsychotic drugs for at least 6 months prior to enrollment. Exclusion criteria comprised:(1) comorbid neurological disorders;(2) active substance abuse or dependence;(3)use of immunomodulators, antioxidants, or anti-inflammatory agents within 12 weeks preceding the study.

All participants received standardized hospital diets with occasional family-supplied snacks (primarily fruits). Daily physical activity was regimented (1 hour of supervised exercise). Most patients were on monotherapy with second-generation antipsychotics, predominantly clozapine and risperidone. Ethical approval was obtained from the institutional Review Board (IRB) of Beijing Hui-Long-Guan Hospital. Written informed consent was secured from all participants or legal guardians.

### Body mass index

Weight and height were measured using calibrated digital scales (SECA 767, Germany) and wall-mounted stadiometers (SECA 217, Germany), respectively. Participants wore light clothing and no shoes. BMI was calculated as weight (kg)/height (m²). According to the criteria of Western Pacific Regional Office of WHO(WPRO), Obesity is defined as BMI ≥25 kg/m² (30), and patients were stratified into high BMI group (BMI ≥25 kg/m²) and low BMI group(BMI <25 kg/m²).

### Psychopathological evaluation

Symptoms were assessed using the 30-item Positive and Negative Syndrome Scale (PANSS), administered by two trained psychiatrists. The PANSS includes three subscales: Positive Symptoms, Negative Symptoms and General Psychopathology. Inter-rater reliability was ensured through standardized training sessions and periodic calibration, achieving an intraclass correlation coefficient (ICC) >0.8 for total PANSS scores.

### Blood sampling

Fasting venous blood samples were collected between 07:00 and 09:00 after an overnight fast (≥8 hours). Serum was separated via centrifugation (3,000 rpm, 15 minutes, 4°C) and stored at −70°C until analysis.

Triglycerides (TG), total cholesterol (TC), high-density lipoprotein cholesterol (HDL-C), and low-density lipoprotein cholesterol (LDL-C) were quantified using enzymatic colorimetric assays (Beijing Leadman Biotechnology Co., Ltd., China) on an Olympus AU2700 autoanalyzer (Japan).

Apolipoproteins (ApoA1, ApoB) and fasting glucose were measured using immunoturbidimetric and hexokinase methods, respectively.

### Cytokine measurements

Serum concentrations of IL-2, IL-6, and TNF-α were determined in duplicate using commercial enzyme-linked immunosorbent assay (ELISA) kits (NeoBioscience Technology, China). Samples were analyzed in a single batch by a technician blinded to clinical data.

We selected only 69 patients out of a total of 201 patients for the following reasons: (1) Funding constraints: Due to budgetary constraints, we randomly selected 100 patients from the 201-patient sample for cytokine testing. (2) Compliance with sample processing and storage protocols: To ensure the reliability of analytical results, we rigorously selected samples that adhered to standard operating procedures (SOPs) throughout the entire process—from collection, centrifugation, aliquoting, to long-term cryopreservation—and showed no signs of repeated freeze-thaw cycles. However, some early-enrolled patients’ samples were excluded due to minor uncertainties in their processing records or storage conditions, which was also a conservative quality assurance approach. (3) Sample quality control (no obvious hemolysis/lipemia): Samples with severe hemolysis or lipemia were excluded. (4) Application of primary exclusion criteria: To minimize confounding factors, particularly the significant impact of recent infections on cytokine levels, we applied the following additional exclusion criteria in the cytokine subgroup: Patients with a documented history of acute infection within 4 weeks prior to enrollment were explicitly excluded (requiring medical record evidence or patient-reported fever, and antibiotic use).

**Figure 1:**
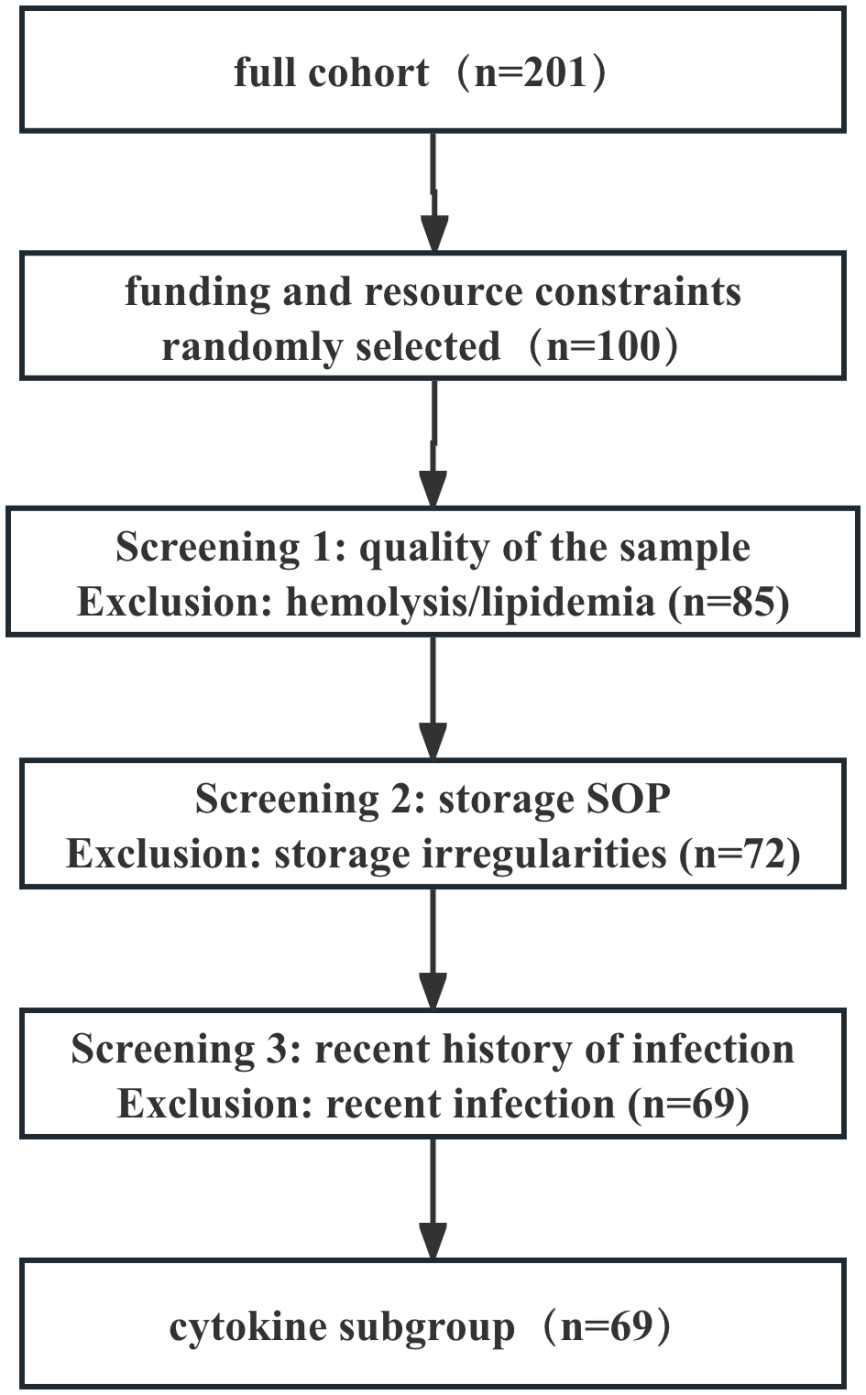
Flowchart for cytokine subgroup screening.

The cytokine subgroup showed no significant differences from the overall group in terms of age, gender, disease duration, age at onset, type and dose of antipsychotic medications. **Figure 1** provides a detailed overview of the screening process.

### Statistical analysis

Data normality was assessed using the Kolmogorov-Smirnov test. Continuous variables were expressed as mean ± SD (normally distributed) or median (interquartile range) (non-normal), while categorical variables were reported as frequencies (%). ANOVA was used to compare normally distributed continuous variables between the high BMI and low BMI groups. *Post-hoc* pairwise comparisons utilized Tukey’ Honestly Significant Difference(HSD)test. Pearson’ correlation coefficient was assessed linear relationships between normally distributed variables. Spearman’s rank correlation coefficient evaluated associations involving non-normal variables. Stepwise linear regression models adjusted for covariates (age, gender, education, illness duration, smoking status, and antipsychotic type) were used to identify independent factors associated with BMI and PANSS subscores. Bonferroni correction addressed multiple testing (*p*<0.05 deemed significant). The presence of multicollinearity among the independent variables was evaluated with the variance inflation factor (VIF), with a VIF > 5 indicating significant multicollinearity.

All analyses were performed using SPSS 25.0 (IBM Corp.,USA). Figures were generated with GraphPad Prism 6.0 (GraphPad Software, USA).

## Results

The study cohort comprised 201 chronic schizophrenia patients, stratified into high BMI and low BMI groups. No significant differences were observed in age, gender, education, age of onset or illness duration. There was no significant difference between the high BMI group and the low BMI group in the proportion of patients using second-generation antipsychotics and first-generation antipsychotics(**χ^2^ =** 0.13, *p*=0.715).However, clozapine use was significantly higher in the high BMI group (*p*= 0.005), aligning with its known metabolic side effects. Additionally, there was no significant difference in the proportion of patients using olanzapine and quetiapine between the two groups.

**Figure 2:**
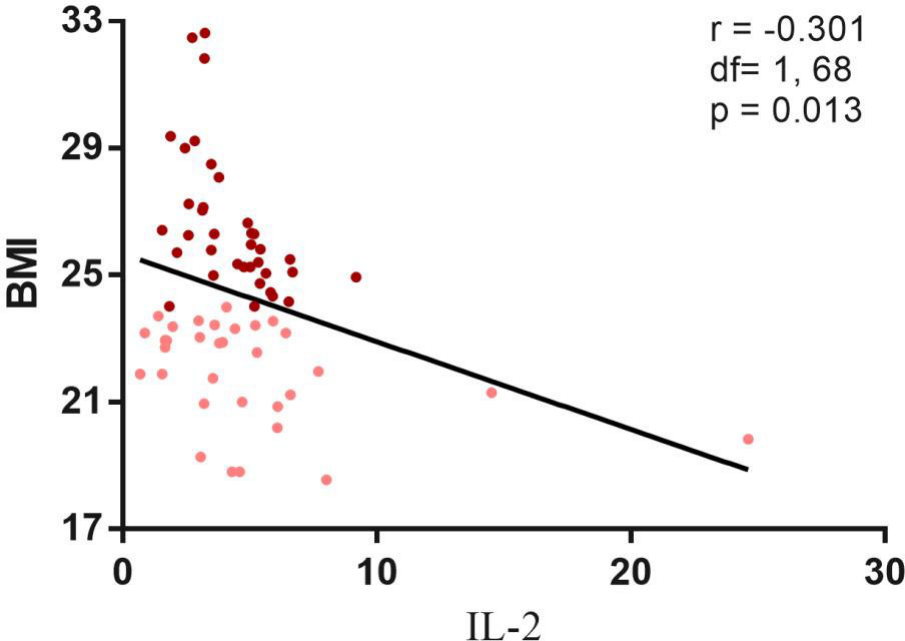
Negative correlation between psychopathology and BMI. Correlation analysis revealed a significantly negative correlation between the BMI and the PANSS negative subscore (r=-0.242, df=1, 200, p=0.001). Maroon dots: high BMI; Pink dots: low BMI; PANSS, Positive and Negative. Syndrome Scale; N, negative psychopathology.

The high BMI group exhibited significantly lower PANSS negative symptom scores compared to the low BMI group (F=5.51, *p*=0.020), even after adjusting for covariates (*p*<0.05). In contrast, PANSS total, positive and general psychopathology scores showed no significant differences (**Table 1**). Correlation analyses further revealed a modest but significant negative association between BMI and PANSS negative subscores (r = −0.242, *p*= 0.001) (**Figure 2**).

**Table 1:** Pyschopathology of chronic schizophrenic patients with/without high BMI.

Psychopathological assessment	All patients (n=201)	High BMI (n=107)	Low BMI (n=94)	F	P	Effect size
PANSS-P	15.51(6.30)	15.24(6.59)	15.82(5.97)	0.42	0.519	0.09
PANSS-N	24.93(6.27)	23.96(5.31)	26.02(7.08)	5.51	0.02	0.34
PANSS-G	33.36(8.62)	32.57(8.32)	34.26(8.90)	1.92	0.167	0.2
PANSS-Total	73.10(16.69)	71.29(15.60)	75.16(17.72)	2.71	0.101	0.23

Patients with higher BMI had lower scores of negative symptoms in PANSS.

The Positive and Negative Syndrome Scale (PANSS).

The high BMI group demonstrated elevated fasting glucose (Z=−2.86, *p*= 0.004), triglycerides (Z=−3.84, *p*<0.001), and apolipoprotein B (Z= −1.99, *p* = 0.047) compared to the low BMI group (**Table 2**). These differences remained significant after covariate adjustment. Spearman correlations confirmed positive associations between BMI and glucose (r= 0.263, *p*<0.001), TG (r= 0.358, *p*<0.001), and LDL-C (r= 0.177, *p*= 0.012).

**Table 2:** Demographic and clinical characteristics of chronic schizophrenic patients with/without high BMI.

Socio-demopraphic and clinical correlates	All patients (n=201)	High BMI (n=107)	Low BMI (n=94)	F/Z/χ²	P
Age, years	51.69(7.83)	51.79(12.68)	51.58(8.06)	0.04	0.081
Female (%)	63(31.34%)	39(36.45%)	24(25.53%)	2.77	0.096
Education, years	9.81(2.48)	9.77(2.32)	9.86(2.67)	-0.18	0.858
Age of onset, years	24.34(6.34)	24.46(6.74)	24.20(5.90)	0.08	0.776
Duration of illness, years	27.33(8.96)	27.19(9.18)	27.48(8.74)	0.05	0.819
Smoking status (smoking/no smoking)	118/83	62/45	56/38	0.06	0.815
BMI, kg/m²	24.40(3.88)	27.30(2.49)	21.11(2.17)	347.87	<0.001
CPZ equivalent, mg/d	385.22(341.83)	379.36(337.70)	391.77(348.27)	-0.15	0.883
Clozapine, %	94(46.76%)	60(56.07%)	34(36.17%)	7.96	0.005
Biochemical parameters					
TG, mmol/L	1.74(1.01)	1.99(1.14)	1.47(0.74)	-3.84	<0.001
HDL-c, mmol/L	1.08(0.16)	1.09(0.15)	1.08(0.16)	0.06	0.812
LDL-c, mmol/L	2.95(0.59)	3.02(0.63)	2.88(0.54)	2.5	0.116
TC, mmol/L	4.45(0.89)	4.53(0.98)	4.36(0.77)	1.92	0.167
ApoA1, mmol/L	1.20(0.15)	1.20(0.10)	1.20(0.19)	-0.27	0.786
ApoB, mmol/L	0.71(0.27)	0.75(0.35)	0.67(0.12)	-1.99	0.047
Glucose, mmol/L	4.88(1.12)	5.05(1.26)	4.68(0.90)	-2.86	0.004

The use of clozapine, levels of TG, ApoB and Glucose were found higher in the high BMI group than the low BMI group.

BMI, Body Mass Index; CPZ, Chlorpromazine; TG, Triglycerides; TC, total cholesterol; HDL-C, high-density lipoprotein cholesterol; LLD-C, low-density lipoprotein cholesterol; CHO, cholesterol; ApoA1, apolipoprotein and ApoB.

**Figure 3:**
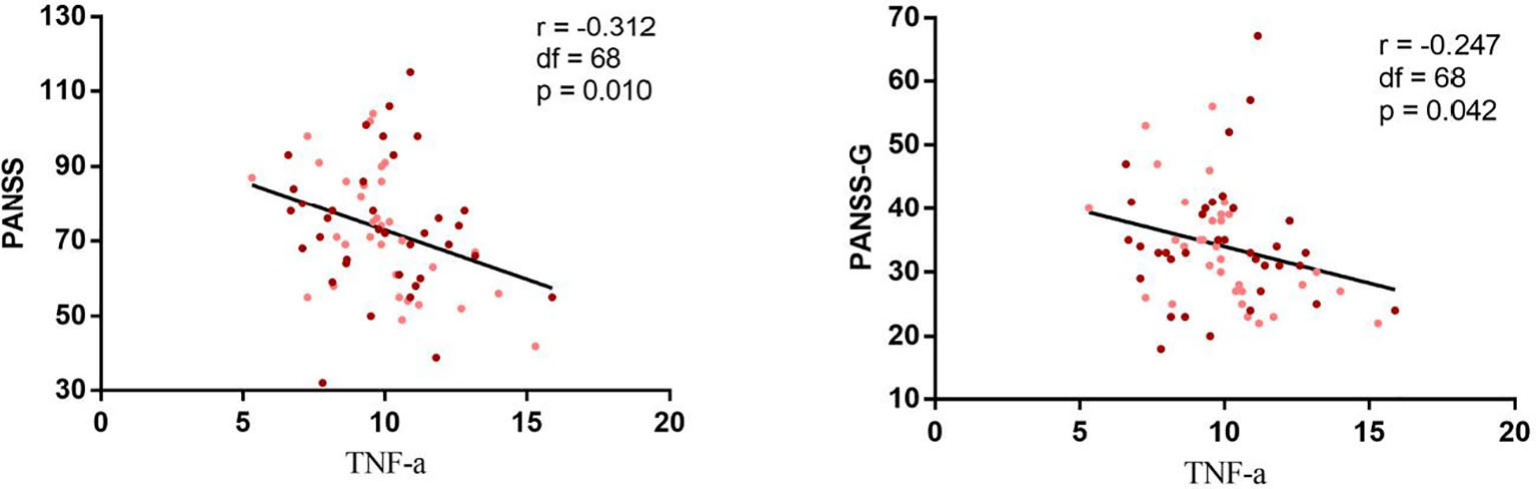
Negative correlation between I correlation between IL-2 and BMI in chronic schizophrenia. Correlation analysis revealed a significantly negative correlation between the II.-2 and BMI in both high r = 0.506 p = 0.002) and low BMI group r = - 0.376 P = 0.034 ) and all patients(r=. 0.301, p = 0.013). Maroon dots: high BMI; Pink dots: low BMI; PANSS, Positive and Negative Syndrome Scale, G, positive psychopathology.

**Figure 4:**
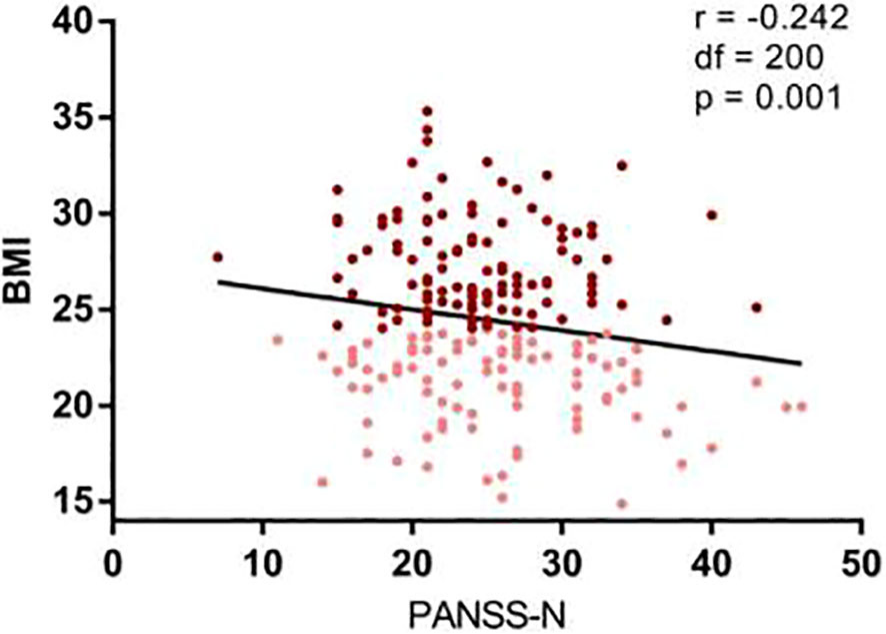
Negative correlations between TNF-alpha and psychopathology of chronic schizophrenia. Correlation analysis revealed a significantly negative correlation between the TNF and the PANSS total score (r = 0.312, df = 1 68, p = 0.01) and PANSS general psychopathology (r 0.247, df = 1 68, P = 0.042) in all patients with schizophrenia. Maroon dots: high BMI, Pink dots: low BMI, PANSS, Positive and Negative Syndrome Scale, G, general psychopathology.

In the cytokine subset, no significant differences in IL-2, IL-6 or TNF-α were observed between BMI groups. However, a significant negative correlation emerged between BMI and IL-2 levels across all patients (r=−0.301, *p*= 0.013) (**Figure 3**). Stratified analyses revealed stronger correlations in the high BMI subgroup (r= −0.506, *p*= 0.002) compared to the low BMI group (r= −0.376, *p*= 0.034). TNF-α levels inversely correlated with PANSS total (r = −0.312, *p*= 0.010) and general psychopathology scores (r = −0.247, *p*= 0.042) (**Figure 4**).

Stepwise regression identified IL-2 (β = −0.236, t= −2.014, *p*= 0.048) and PANSS negative subscore (β = −0.236, t = −3.396, *p*= 0.001) as independent factors associated with lower BMI, explaining 14.1% and 5.6% of variance, respectively. Clozapine use also contributed to higher BMI (β = 0.292, t= 2.492, *p*= 0.015), accounting for 19.4% of variance.

TNF-α was independently associated with lower PANSS general psychopathology scores (β= −0.515, t=−3.294, *p*=0.003), accounting for 24.1% of variance. No significant associations were found between cytokines and lipid/glucose parameters.

## Discussion

The present study elucidates novel associations between obesity-related metabolic dysregulation, cytokine profiles, and psychopathological manifestations in patients with chronic schizophrenia. Our findings highlight three key observations: (1) an inverse relationship between body mass index (BMI) and interleukin-2 (IL-2) levels, independent of antipsychotic exposure; (2) attenuated negative symptoms in patients with higher BMI; (3) a paradoxical negative correlation between tumor necrosis factor-alpha (TNF-α) levels and psychopathology severity. These results challenge conventional paradigms of inflammation in schizophrenia and underscore the complexity of immune-metabolic interactions in this population.

The significant inverse correlation between IL-2 and BMI contrasts with findings in general populations, where obesity is typically associated with elevated proinflammatory cytokines (14, 15).But there was also a study found that individuals with obesity show significantly decreased levels of serum IL-27 (31).This discrepancy may reflect unique pathophysiological mechanisms in schizophrenia, potentially involving antipsychotic-induced immunomodulation. Preclinical evidence suggests that IL-2 deficiency impairs lipid metabolism and adipocyte differentiation (32), while genetic polymorphisms in IL-2 have been linked to antipsychotic-induced weight gain (20). Our observation that IL-2 was independently associated with lower BMI after adjusting for clozapine use and illness duration raises the possibility of IL-2 as a compensatory anti-inflammatory mediator in chronic metabolic stress. However, the absence of IL-6-BMI associations in our cohort diverges from meta-analyses in non-psychiatric populations (15, 33), emphasizing the need for disease-specific biomarker frameworks.

The reduced PANSS negative symptom scores in the high BMI group align with prior reports of inverse BMI-negative symptom correlations (34, 35). While speculative, this phenomenon could reflect improved social engagement due to structured hospital diets or neuroprotective effects of adipokines such as leptin. Conversely, the lack of association between BMI and positive symptoms suggests distinct neurobiological pathways mediating different symptom domains. The negative correlation between TNF-α and PANSS general psychopathology scores further complicates the inflammatory hypothesis of schizophrenia. Previous studies have reported inconsistent findings regarding the relationship between TNF-α levels and psychiatric symptoms. Some studies found no significant correlation between TNF-α levels and PANSS total scores or subscale scores (36, 37), while others reported a negative correlation between TNF-α levels and PANSS total scores, P subscale, or G subscale (38, 39), or a positive correlation with the N subscale (40). These differences may be influenced by various factors. First, TNF-α levels are influenced by factors such as age, gender, smoking, and BMI (41). Second, differences in methods of measuring TNF-α, recruiting patients at different stages or with different types of schizophrenia, and using different antipsychotic medications may also affect study results (37). Additionally, multiple cytokines form a complex system with the central nervous system, and the role of TNF-α as a single indicator within this cytokine system requires further exploration (42).

Although elevated TNF-α is implicated in acute exacerbations (18), its suppression in chronically medicated patients (38) may reflect long-term antipsychotic effects, as clozapine is known to modulate cytokine production (27).

Our results conflict with studies reporting positive correlations between obesity and IL-6/TNF-α in schizophrenia (26, 43). These inconsistencies may stem from differences in sample characteristics and methodological approaches. Notably, the absence of IL-6-BMI associations in our cohort contrasts with meta-analytic findings in general populations (15), suggesting that schizophrenia-specific factors, such as prolonged antipsychotic exposure or genetic susceptibility, may alter canonical obesity-inflammation pathways.

Several limitations of this study should be acknowledged. First, the absence of a healthy control group precludes the ability to determine the specificity of the observed findings to schizophrenia, as opposed to other psychiatric or general medical conditions. Including healthy controls in future studies would help clarify whether the identified associations are unique to schizophrenia or reflect broader inflammatory or metabolic dysregulation. Second, the sample size, particularly for the subgroup with cytokine measurements was relatively small, which may limit the statistical power and generalizability of the results. Larger, well-powered cross-sectional and longitudinal studies are needed to validate these findings and explore potential causal relationships between cytokines, obesity, and psychopathology. Third, the study assessed only a limited panel of cytokines, which restricts the ability to capture the full spectrum of immune-inflammatory activity. A more comprehensive evaluation of additional cytokines, chemokines, and anti-inflammatory markers would provide a more nuanced understanding of the complex interplay between immune dysregulation, metabolic dysfunction, and psychiatric symptoms. Fourth, this study used body mass index (BMI) to assess obesity status. Although BMI is a commonly used indicator for obesity screening and classification, it does not reflect body fat distribution. However, abdominal obesity is a stronger predictor of metabolic syndrome. Since we did not measure patients’ waist circumference and hip circumference, we were unable to assess the prevalence of abdominal obesity and its impact. Fifth, although this study adjusted for the type of antipsychotic drugs, the lack of standardized dose conversion (e.g., chlorpromazine equivalent dose) weakened control over drug efficacy. Due to the limitations of the study, we were unable to collect antipsychotic drug dosages in this study. We will collect both the types and dosages of antipsychotic drugs in future similar studies to address this shortcoming in this study. Sixth, This study only examined the relationship between IL-6, TNF-α, and IL-2 levels and psychiatric symptoms and obesity, without testing other factors, particularly C-reactive protein (CRP) and interleukin-10 (IL-10). CRP is a commonly used inflammatory biomarker, and previous studies have confirmed that CRP levels can influence metabolic syndrome and schizophrenia symptoms (44). Additionally, CRP levels are significantly correlated with cytokine levels such as IL-6 and TNF-α (45). Furthermore, IL-10 is also closely associated with schizophrenia and metabolic syndrome (17). This is indeed a limitation of this study, which should be addressed in future research by employing a comprehensive cytokine detection panel to compensate for the limitations of this study. Finally, we did not control for some confounding variables adequately in this study. In this study, the patients had been taking heterogeneous antipsychotics, doses and long-term treatment. Numerous studies have reported that treatment with antipsychotic drugs may have immunosuppressive effects and affect the cytokine network. Furthermore, a range of cytokine alterations has been found in acute and chronic schizophrenic patients treated with antipsychotic drugs (46). Although we did not find that the type of antipsychotic drugs was associated with IL-2, IL-6, and TNF-α levels this study, we did not collect the data regarding antipsychotic doses and duration of antipsychotic treatment. Some studies have well-documented dose-dependent effects on cytokines/metabolism (47, 48). In addition, several confounding factors that may influence metabolism or inflammatory processes —such as frequency of family-supplied snacks, number of cigarette smoking, dietary patterns, physical activity levels, and socioeconomic status—were not systematically collected or adjusted for in the analyses. These unmeasured variables could potentially bias the observed associations and should be addressed in future investigations. Given these limitations, the current findings should be interpreted as preliminary. The intricate relationship between cytokines, obesity, and psychopathology in schizophrenia necessitates further confirmatory studies with larger, more diverse cohorts, longitudinal designs, and comprehensive assessments of immune, metabolic, and lifestyle factors. Such efforts will be critical for elucidating the underlying mechanisms and informing targeted therapeutic interventions.

In summary, our findings underscore the intricate interplay between obesity, inflammation, and psychopathology in chronic schizophrenia, challenging simplistic models of “pro-inflammatory” or “anti-inflammatory” states. While IL-2 and TNF-α emerge as potential biomarkers, their roles in disease progression remain enigmatic. A precision medicine approach—integrating immune profiling, genetic data, and clinical phenotypes—is essential to unravel these complexities and improve outcomes for this vulnerable population.

## Data Availability

The raw data supporting the conclusions of this article will be made available by the authors, without undue reservation.
