# Supplementary material for: Obesity, cytokines and psychopathology in patients with chronic schizophrenia
Source: Front Psychiatry. 2025 Jul 28;16:1574041. doi: 10.3389/fpsyt.2025.1574041 (PMC12336245; doi:10.3389/fpsyt.2025.1574041)
Supplement: Supplementary file 1 [file Table1.doc]

**精神分裂症代谢、认知功能**

**调查研究记录手册**

患者姓名：▁▁▁▁▁▁▁▁

患者病区：▁▁▁▁▁▁▁▁

研究医院：▁▁▁▁▁▁▁▁

住 院 号：□□□□□□□□

评 定 者：▁▁▁▁▁▁▁▁

评定时间：▁▁▁▁▁▁▁▁

| 性别： □男 □女 |
| --- |
| 年龄： □□ 岁 |
| 受教育年限：□□ 年 （注：小学＝5年；初中＝8年；中专/高中＝11年；大学本科＝15年） |
| **精神分裂症：请在诊断亚型上画圈**  解体型：295.10 紧 张 型：295.20 偏执型：295.30  残留型：295.60 未分化型：295.90  分裂样障碍：295.40 分裂情感性障碍：295.70 |
| **首次精神症状发生年龄：**□□ 岁 **首次住精神病院年龄：** □□ 岁 |
| **总病程：** _______年 ________ 月 **住院次数：**_______________ 次 |
| **血压：** / mmHg  **身高：**________厘米;  **体重相关资料**  首次住精神病院的体重：_______________公斤  目前体重：_______________公斤  目前腰围：______________ 厘米  目前臀围：_______________厘米 |

| **目前正在服用的主要抗精神病药或抗抑郁药：**  1．名称 剂量： ；用药时间：___________月  2．名称 剂量： ；用药时间：___________月  3．名称 剂量： ；用药时间：___________月  **既往曾使用过主要抗精神病药或抗抑郁药：**  1．名称 剂量： ；用药时间：___________月  2．名称 剂量： ；用药时间：___________月  3．名称 剂量： ；用药时间：___________月 |
| --- |
|  |

**糖尿病、高血压、高脂血症家族史：**

1、糖尿病家族史：A、无 B、有，请具体说明

2、高血压家族史：A、无 B、有，请具体说明

3、高脂血症家族史：A、无 B、有，请具体说明

**吸烟相关资料收集**

**吸烟调查**

**（一）、不吸烟：**从没吸烟，或吸烟少于100支/终生

**（二）、过去曾吸烟**（吸烟多于1支/每天，时间多于1年），现在已戒烟；

1．您开始长期吸烟的年龄：

2．最初尝试吸烟的原因：a发现吸烟能改善自己的病情；b模仿他人；

c他人诱惑； d交际需要； e其它_________ __

3．您上次吸烟离现在多长时间：_________

4．您在戒烟前，共吸烟多长时间：________；每天平均吸烟大约________支；

5．您曾经戒烟多少次：___________次；

6．戒烟原因：主动意愿 □a担心身体健康；b认识到吸烟的危害；c其它____ __

被动戒烟 □ a环境所限如无烟病房；b躯体疾病；c 家人 要求； d经济负担；

e其它______ ________

7．最近一次戒烟的方法：

a骤然停止；b逐渐递减；c药物治疗；d其它___ __

8．您戒烟是否失败过？□否 □是

最近一次戒烟失败的原因：a心理渴求；b他人诱惑； C戒断症状；d自觉病情加重；

e其它___ ____

**（三）、目前吸烟**（吸烟多于1支/每天，时间多于1年）

1．您开始吸烟的年龄：□□岁

2．您多长时间后开始规律吸烟（每天至少一支）：□□□月

3．最初尝试吸烟的原因：a发现吸烟能改善自己的病情_______ ____；b模仿他人；

c他人诱惑； d交际需要； e其它___________

4．您目前平均每天吸多少支烟 ___________支（近1周内）；

5．您得病前是否吸烟？ 否

是 平均每天吸多少支烟 __________支（病前1周）；

6．您首次住院前是否吸烟？ 否

是 平均每天吸多少支烟 ________支（病前一周）；

7．您吸烟最多时，在一周的时间里，平均每天吸_________支；

8．您曾戒过烟吗？ 否

是 戒过几次_______；戒烟最长时间___________月。

9．戒烟原因：主动意愿 □a担心身体健康；b认识到吸烟的危害；c其它______

被动戒烟 □a环境所限如无烟病房；b躯体疾病； c家人要求；

d经济负担；e其它_________ _____

10．最近一次戒烟的方法：a骤然停止；b逐渐递减；c药物治疗；d其它_ ____

11．最近一次戒烟失败的原因：a心理渴求；b他人诱惑；c戒断症状；

d自觉病情加重；e其它 _______

**（四）、家人吸烟情况**

1．您父亲吸烟：否 是（平均_____支/日）；您母亲吸烟：否 是（平均_____支/日）

2．圈出其他所有长期吸烟者：爷爷、奶奶、外祖父、外祖母、哥哥、弟弟、姐姐、

妹妹、叔叔、舅舅、姑姑、姨

3．他们吸烟支数：家人最多吸烟______支/日；最少______支/日；中间______支/日。

**尼古丁依赖严重程度量表（FNTD）**

| 问题 | 答案 | 分数 |
| --- | --- | --- |
| 1你通常在起床后多长时间吸第一支烟？ | 5分钟以内 | 3 |
| (你通常在起床后多长时间想吸第一支烟？) | 6—30分钟 | 2 |
|  | 31—60分钟 | 1 |
|  | 1小时以后 | 0 |
| 2在不准吸烟的场所你感到受限制吗？ | 是 | 1 |
|  | 否 | 0 |
| 3如果在一天中你必须取消一次吸烟的机 | 晨起后的第1支 | 1 |
| 会，你不愿意取消哪一次？ | 一天中任何一支 | 0 |
| (如果在一天三次的吸烟机会中你必须取消一次吸烟的机会，你不愿意取消哪一次？) |  |  |
| 4你每天吸几支香烟？ | 多于30支 | 3 |
|  | 21—30支 | 2 |
|  | 11—20支 | 1 |
|  | 少于10支 | 0 |
| 5清晨醒来后的1小时内的吸香烟数量比其他时间1小时内 | 是 | 1 |
| 吸烟数量多吗？ | 否 | 0 |
| (早餐后第一次吸烟是否会比其它两次吸烟量多？) |  |  |
|  |  |  |
| 6即使因为不舒服而必须躺在床上也要吸烟吗？ | 是 | 1 |
| (即使身体不舒服，也必须要使自己在指定时间到指定地点 | 否 | 0 |
| 吸烟吗？) |  |  |

**0-2**分：很低；**3-4**分：低；**5**分：中等程度；**6-7**分：重度；**8-10**分：极重度。 总分：______。

饮酒相关资料收集

| **喝酒情况**（指患者在家里时的情况） |
| --- |
| 一、发病前：□ |
| 1＝从不饮酒；2＝轻度；3＝中度；4＝重度；5＝严重 |
| 二、发病后的任何时期：□ |
| 1＝从不饮酒；2＝轻度；3＝中度；4＝重度；5＝严重 |

（说明：每周按7天计算, 饮酒严重程度如下：

轻度：平均每周饮酒1--3次，每次大约相当于半两白酒或250ml啤酒或50ml 葡萄酒；（说明：偶尔喝酒一次不算，必须每周都固定饮酒）；

中度： 平均每周饮酒5次左右，每次大约相当于1两白酒，或500ml啤酒，或100ml 葡萄酒；

重度：平均每周饮酒10次左右，每次大约相当于2两白酒, 或500ml啤酒，或200ml 葡萄酒；

（说明：如果每周有两次在早晨空腹时就要饮酒，不管每周饮酒次数，每次饮酒量，都是重度）。

严重：平均每周饮酒13以上， 每次大约相当于3两白酒, 或750ml啤酒，或300ml 葡萄酒；

（说明：如果每周有三次以上在早晨空腹时就要饮酒，不管每周饮酒多少次，每次饮酒量多少，都是严重）。

**糖尿病情况**

**一、是否被诊断为“糖尿病”**

1、是： A. 采用降糖治疗； B.没有治疗

2、否

3、若选“1”，请问患者何时被诊断为糖尿病：

A、首次发病前

B、首次发病后

C、使用抗精神病药物以前

D、使用抗精神病药物以后

（在以上四个选项中选出两项，其中A、B项任选其一，C、D项任选其一）

**二、请从病历中找出病人最近一次血糖浓度：**

Glu: _____________________________, 检测日期_______ 年______月 ______ 日

**脂代谢情况：**

**一、是否被诊断为“高脂血症”**

1、是： A. 采用降脂治疗； B.没有治疗

2、否 降脂药物使用情况_______________________

3、若选“1”，请问患者何时被诊断为高脂血症：

A、首次发病前

B、首次发病后

C、使用抗精神病药物以前

D、使用抗精神病药物以后

（在以上四个选项中选出两项，其中A、B项任选其一，C、D项任选其一）

**高血压情况：**

**一、是否被诊断为“高血压”**

1、是： A. 采用降压治疗； B.没有治疗

2、否

3、若选“1”，请问患者何时被诊断为高血压：

A、首次发病前

B、首次发病后

C、使用抗精神病药物以前

D、使用抗精神病药物以后

（在以上四个选项中选出两项，其中A、B项任选其一，C、D项任选其一）

**二、请从病历中找出病人最近一次血压检测结果：**

收缩压/舒张压 __________________ 检测日期_______ 年______月 ______ 日

**内分泌情况：**

**请从病历中找出病人最近一次泌乳素检测结果：**

泌乳素 __________________ 检测日期_______ 年______月 ______ 日

**最近一次心电图：**

| 心电图  （ECG） | 心率 | QRS宽 | PR间期 | QT间期 | 总印象 |
| --- | --- | --- | --- | --- | --- |
|  | 次/分 |  |  |  |  |

注：ECG总印象编号：0=正常 1=异常但无临床抑郁 2=异常有临床意（请具体注明： ）

检测日期：_________年_________月_________日

**目前是否合并其它诊断（包括精神和躯体两方面）：**

1. 是 2、否

若选择“是”，请列出：

1. __________________________________________________
2. __________________________________________________
3. __________________________________________________

4）__________________________________________________

**既往史：**

1、有 2、无

若选择“有”，请列出：

1）__________________________________________________

2）__________________________________________________

3）__________________________________________________

4）__________________________________________________

**阴性与阳性症状量表（PANSS）**

说明：请圈出相应的分数

| 无 极轻 轻度 中度 偏重 重度 极重 |
| --- |
| **1）阳性分量表** P1 妄想 1 2 3 4 5 6 7 P2 *概念紊乱 1 2 3 4 5 6 7  P3 *幻觉行为 1 2 3 4 5 6 7  P4 *兴奋 1 2 3 4 5 6 7  P5 *夸大 1 2 3 4 5 6 7  P6 *猜疑或被害 1 2 3 4 5 6 7  P7 *敌对性 1 2 3 4 5 6 7  分量表分（ ）  **2）阴性分量表** N1 *情感迟钝 1 2 3 4 5 6 7 N2 *情绪退缩 1 2 3 4 5 6 7  N3 情感交流障碍 1 2 3 4 5 6 7  N4 被动或淡漠 1 2 3 4 5 6 7  N5 抽象思维 1 2 3 4 5 6 7  N6 交谈缺乏自发性和流畅性 1 2 3 4 5 6 7  N7 刻板思维 1 2 3 4 5 6 7  分量表分（ ）  **3）一般精神病理学分量表**  G1 *担心身体健康 1 2 3 4 5 6 7  G2 *焦虑 1 2 3 4 5 6 7  G3 *罪恶观念 1 2 3 4 5 6 7  G4 *紧张 1 2 3 4 5 6 7  G5 *装相和作态 1 2 3 4 5 6 7  G6 *抑郁 1 2 3 4 5 6 7  G7 *动作迟缓 1 2 3 4 5 6 7  G8 *不合作 1 2 3 4 5 6 7  G9 *异常思维内容 1 2 3 4 5 6 7  G10 *定向障碍 1 2 3 4 5 6 7  G11 注意障碍 1 2 3 4 5 6 7  G12 自知力缺乏 1 2 3 4 5 6 7  G13 意志障碍 1 2 3 4 5 6 7  G14 冲动控制障碍 1 2 3 4 5 6 7  G15 先占观念 1 2 3 4 5 6 7  G16 主动社交回避 1 2 3 4 5 6 7  分量表分（ ）  **PANSS总分（ ） BPRS总分（ ）**带*项目的和 |

**Hamilton 抑郁症状量表 (HAMD)**

| 项目 | 评分 | 项目 | 评分 |
| --- | --- | --- | --- |
| 忧郁情绪 | 0 1 2 3 4 | 性症状 | 0 1 2 |
| 有罪感 | 0 1 2 3 | 疑病 | 0 1 2 3 4 |
| 自杀 | 0 1 2 3 4 | 体重减轻 | 0 1 2 |
| 入睡困难 | 0 1 2 | 自知力 | 0 1 2 |
| 睡眠不深 | 0 1 2 | 日夜变换：A早  日夜变换：B晚 | 0 1 2 |
| 早醒 | 0 1 2 |  | 0 1 2 |
| 工作和兴趣 | 0 1 2 3 4 | 人格或现实解体 | 0 1 2 3 4 |
| 阻滞 | 0 1 2 3 4 | 偏执症状 | 0 1 2 3 4 |
| 激越 | 0 1 2 3 4 | 强迫症状 | 0 1 2 |
| 精神焦虑 | 0 1 2 3 4 | 能力减退感 | 0 1 2 3 4 |
| 躯体焦虑 | 0 1 2 3 4 | 绝望感 | 0 1 2 3 4 |
| 胃肠道症状 | 0 1 2 | 自卑感 | 0 1 2 3 4 |
| 全身症状 | 0 1 2 | **总分** |  |

**汉密尔顿焦虑量表（HAMA）**

| **项 目** | | **评分**^*^ |
| --- | --- | --- |
| 1.焦虑心境 | 担心，预感会发生最坏的情况，恐惧性的期盼，易激惹 | □ |
| 2.紧张 | 紧张、疲劳、惊恐反应，易激动，颤抖，不能平静，不能放松 | □ |
| 3.害怕 | 怕黑暗，怕生人，怕独自一人，怕动物，怕过马路，怕人多拥挤 | □ |
| 4.失眠 | 难入睡，睡眠中断，睡眠不足或醒后感觉困乏，多梦、夜惊 | □ |
| 5.记忆或注意  障碍 | 注意力不集中，记忆力不好 | □ |
| 6.抑郁心境 | 缺乏兴趣，对各项业余爱好感到索然无味，抑郁、早醒，一日之  内心境有波动 | □ |
| 7.躯体性焦虑  （肌肉系统） | 疼痛、抽搐、强直、磨牙、说话声音颤抖、音调增高 | □ |
| 8.躯体性焦虑  （感觉系统） | 耳鸣，视物模糊，忽冷忽热感，体弱感，刺痛感 | □ |
| 9.心血管系统  症状 | 心动过速、心悸，胸痛，血管波动感，发昏，心律不齐 | □ |
| 10.呼吸系统  症状 | 胸部压迫，呼吸不畅，叹气，呼吸困难 | □ |
| 11.胃肠道症状 | 吞咽困难，多排气，腹痛，腹部灼热感，腹胀，恶心，呕吐，肠  鸣，大便溏稀，体重减轻，便秘 | □ |
| 12.生殖泌尿  系统症状 | 尿频、尿急，闭经、月经过多，性冷淡，早泄，性欲缺乏，阳萎 | □ |
| 13.植物神经  系统症状 | 口干，面色潮红，面色苍白，易出汗，头晕目眩，紧张性头痛，  竖毛 | □ |
| 14.会谈时  行为表现 | 烦躁不安，坐立不安或来回走动，手发抖，皱眉，绷脸，叹息或  呼吸急促，面色苍白，吞咽，嗳气，瞳孔扩大，突眼 | □ |
| **总 分** | | □□ |

^*^评分标准：0=无症状；1=轻；2=中等；3=重；4=极重。

**Hoffman幻听量表**

患者是否存在幻听 □是（请评定幻听量表） □否（跳过）

1. 每天出现幻听的频率：□

0=无； 1=极少出现（每天1-5次）；

2=偶尔出现（每天6-10次）； 3=较少出现（每小时1-2次）；

4=经常出现（每小时3-6次） 5=频繁出现（每小时7-10次）；

6=非常频繁出现（每小时11-20次） 7=极度频繁出现（每小时21-50次）

8=快速反复出现（每分钟1次）； 9=持续存在

1. 病人对幻听的感觉（真实性）：□

0=与思维难以区分； 1=假想的； 2= 声音模糊

3=梦境般体验； 4=比较真实； 5=非常真实

1. 与普通说话声相比，幻听声音的大小/强弱：□

0= 声音轻微，听不清； 1=声音低，但可以听清； 2=声音柔和

3=与普通说话声相当； 4=类似于大声说话； 5=与喊叫或尖叫声相当

1. 幻听中可辨别出声音的种类：□

0=无； 1=1种； 2=2种；

3=3种； 4=4种 5=5种； 6=5种以上

1. 幻听主要内容的长度（言语性幻听）：□

0=非言语性幻听； 1=以单个字出现； 2=以词汇的形式出现

3=以完整的简单句出现； 4= 以复杂的句型出现

1. 病人对幻听注意程度：幻听对病人思维、情感和行为的影响：□

1=无影响； 2= 偶尔影响； 3= 当声音出现时暂时受影响

4=声音出现时通常影响患者的注意力；

5= 幻听经常影响病人的思维、语言和行为

6=幻听完全影响病人的思维、情感和行为（如与幻听对话、发笑）

7=病人的思维、情感和行为完全受幻听的支配

1. 幻听给病人带来的痛苦程度：□

1=无痛苦体验； 2=轻度痛苦； 3=中度痛苦

4=有时产生明显焦虑； 5=经常产生明显的恐惧或焦虑

**异常不自主运动评定量表（AIMS）**

| **项目 评 分** |
| --- |
| 1、表情肌 0 1 2 3 4  2、唇及口周 0 1 2 3 4  3、颌 0 1 2 3 4  4、舌 0 1 2 3 4  5、上肢 0 1 2 3 4  6、下肢 0 1 2 3 4  7、颈、肩、髋 0 1 2 3 4  8、异常运动的严重程度 0 1 2 3 4  9、因异常运动的而致能力丧失 0 1 2 3 4  10、病人对异常运动的察觉 0 1 2 3 4  11、牙齿和假牙问题 0 1 2 3 4  12、是否常戴假牙 0 1 2 3 4 |

**AIMS总分：_____________**

**锥体外系副反应量表（RSESE）**

| **项目 评分** |
| --- |
| 1、步态 0 1 2 3 4  2、落臂 0 1 2 3 4  3、摇肩 0 1 2 3 4  4、肘强直 0 1 2 3 4  5、固定姿势或腕强直 0 1 2 3 4  6、腿的摆动 0 1 2 3 4  7、头颈部运动 0 1 2 3 4  8、眉间点敲 0 1 2 3 4  9、震颤 0 1 2 3 4  10、流涎 0 1 2 3 4  **RSESE**总分：**_____________** |

**Simpson锥体外系副反应评定量表（SEPS）**

**1步态**

正常………………………………………..…………………………………………0

病人步行时双臂摆动减少……………………..…………………………………..1

病人步行时双臂摆动明显减少……………………..……………………………..2

僵直无摆动………………………………………………..…………………………3

僵直而拖着脚步走动…………………………………………..…………………..4

**2平衡**

正常……..…………………………………………………………………………..0

可疑的异常…..……………………………………………………………………..1

肯定的异常……..…………………………………………………………………..2

**3落臂动作**

正常…………………..……………………………………………………………..0

触及时不明显……………..………………………………………………………..1

无反弹动作……………………..…………………………………………………..2

无“啪”的声响………………………………………………………………………3

好象遇到阻力一样………………………..………………………………………..4

**4大关节和动作僵硬**

正常……………………………………………..…………………………………..0

轻度僵硬……………………………………………..……………………………..1

中度的僵硬；肯定的异常………………………………..………………………..2

重度的僵硬；但较易达到整个动作的幅度………………………………………..3

极重度的僵硬；整个动作幅度受限或难以达到…………………..……………..4

**5齿轮样肌肉僵直**

无…………………..…………………………………………………………………0

很轻或可疑……………..…………………………………………………………..1

肯定存在……………………..……………………………………………………..2

**6点触眉间**

正常………………………………..………………………………………………..0

可疑的异常……………………………..…………………………………………..1

明显的异常…………………………………..……………………………………..2

**7震颤**

没有或难以看出……..……………………………………………………………..0

轻度；仅在伸出上肢做诱发时存在……..………………………………………..1

中度；静止时持续存在，幅度较明显………..…………………………………..2

明显的持续存在且幅度较大………………………..……………………………..3

极重度：有肯定的功能受损……………………………..………………………..4

**8运动不能**

无………………………………………………………………..…………………..0

轻度：动作缓慢，有一种故意做作的感觉或言语平淡且自发言语减少……....1

中度：运动缺乏，很少有表达性姿势或自发性言语……………………………..2

重度：动作或言语始动困难，连续动作受阻……………………………………..3

极重度：需作出很大努力才能运动或呆滞不动，缄默不语…………………....4

**9静坐不能**

无……………………………………………………………………………………..0

轻度：检查时偶尔可见静止不能和/或自述肯定有…………………..………...1

中度：可见持续的静止不能和/或自述有明显的烦躁不安…………………..….2

重度：检查时病人不停的站起和坐下，难以集中注意力……………………....3

极重度：动作行为明显增多；惊恐不安…………………………..……………..4

**10急性肌张力障碍**

无……………………………………………………………………………..……..0

有，描述 ………………..……………………………………..1

**11合作性**

合作……………………………………………………………………………………0

有轻度的阻抗，但能执行检查要求………………………………………………..1

不合作，完成检查时有很大的困难或有一两项尚不能完成………..…………..2

十分不合作，不能完成多项检查项目………………………………………..…..3

# **Barnes静坐不能量表**

| **1、客观** | 0＝正常，偶尔有肢体活动的僵硬；  1＝存在特征性的不安性运动：在坐位时腿脚的曳行或踏步运动或一条腿摆动，  在站立时两脚来回摆动或原地踏步，但这些运动存在的时间少于观察时间的一半；  2＝如上述（1）条所述的现象存在的时间占到观察时间的至少一半；  3＝病人持续存在特征性的不安性运动，和／或病人在被观察期间不能保持坐  位或站位而不走动或踏步 |
| --- | --- |
| **2、主观- 对多**  **动不安的知觉** | 0＝不存在内在的不安感；  1＝非特异性的内在不安感；  2＝病人感觉到不能保持腿的静止，或感到对腿部活动的渴求，和／或诉说若  要求其静止站立，则会特定地出现内心不安感的增强；  3＝感到大多数时间有一种强烈地要求活动的冲动，和／或诉说在多数时间里  有强烈地想走动或踏步的要求 |
| **3、主观-与不安相关的痛苦** | 0＝无痛苦；1＝轻度；2＝中度；3＝重度 |
| **4、对静坐不能**  **的总体临床评价** | 0＝无；无感觉到的不安的证据。观察到的特征性的静坐不能运动，若没有主  观报告的内心不安感或活动腿部的冲动性渴求，应归为假性静坐不能；  1＝可疑；非特异性内在紧张感和运动僵硬；  2＝轻度静坐不能；感觉到腿部的不能静止以及内心的不安感在被要求静止站  立时会加重。存在僵硬的运动，但不一定能观察到特征性的不能静止的静  坐不能性运动；  3＝中度静坐不能；感觉到上述出现于轻度静坐不能的不安感，伴有特征性的  不安运动，如在站立时两脚来回摆动。病人因这种情况感到痛苦；  4＝显著静坐不能；主观感到不安，包括对走动或踏步的冲动性渴求。但病人  能够保持坐位至少5分钟。此状态显然是痛苦的；  5＝严重静坐不能；病人报告绝大多数时间存在强烈地想要上下踏步的冲动。  不能坐下或躺下数分钟。持续性不能静止并伴有强烈的痛苦和失眠 |

| **自杀行为或自杀观念**……□  1＝从没有过自杀行为或自杀观念；  2＝曾经有过自杀观念，但从未付诸行动；  3＝曾经自杀未遂；  ***若选3，请继续回答：***  ·自杀未遂几次： □□次  ·大约在什么时间： □□□□年□□月 （最近一次） |
| --- |

**认知测查**

**RBANS** 是否完成RBANS测查 □是 □否

**RBANS评定测查结果**

| 即刻记忆 | □□□ |
| --- | --- |
| 视觉广度 | □□□ |
| 言语功能 | □□□ |
| 注意力 | □□□ |
| 延迟记忆 | □□□ |
| 总分 | □□□ |

**Stroop**  是否完成Stroop测查 □是 □否

**STROOP评定检测结果：**

| 单字错误数 | 单字时间 | 单色错误数 | 单色时间 | 双字错误数 | 双字时间 | 双色错误数 | 双色时间 |
| --- | --- | --- | --- | --- | --- | --- | --- |
|  |  |  |  |  |  |  |  |

**神经影像学检测 MRI** 是 否

**电生理检测**  是 否

**生物学指标检测：**取清晨空腹学10ml, 分离血清、血浆。

**实验室检查记录**

**日期___________________**

| **血**  **常**  **规** | 白细胞(WBC) | | 10^*9^/L | | | | | | |
| --- | --- | --- | --- | --- | --- | --- | --- | --- | --- |
|  | 淋巴细胞百分比（LYM%） | | % | | | | | | |
|  | 单核细胞百分比（MONO%） | | % | | | | | | |
|  | 中性粒细胞百分比（NEUT%） | | % | | | | | | |
|  | 红细胞（RBC） | | 10^*12^/L | | | | | | |
|  | 血红蛋白（HGB） | | g/L | | | | | | |
|  | 血小板（PLT） | | 10^*9^/L | | | | | | |
|  | 其它 | |  | | | | | | |
| **血**  **生**    **化** | 谷丙转氨酶（ALT） | | u/L | | 血糖（GLU） | | mmol/L | | |
|  | 总蛋白（TP） | | g/L | | 钙（Ca） | | mmol/L | | |
|  | 白蛋白（ALB） | | g/L | | 总胆固醇（CHO） | | mmol/L | | |
|  | 球蛋白（GLO） | | g/L | | 甘油三酯（TG） | | mmol/L | | |
|  | 总胆红素（T-BIL） | | umol/L | | 高密度脂蛋白  胆固醇（HDL） mmol/L | | | | |
|  | 谷氨酰转肽酶（GGT） | | u/L | |  |  |  |  |  |
|  | 谷草转氨酶（AST） | | u/L | | 低密度脂蛋白  胆固醇（LDL） mmol/L | | | | |
|  | 乳酸脱氢酶（LDH） | | u/L | |  |  |  |  |  |
|  | 肌酸激酶（CPK） | | u/L | | 载脂蛋白A1（APOA1） | | | g/L | |
|  | 血淀粉酶（AMY） | | u/L | | 载脂蛋白B（APOB） | | | g/L | |
|  | 胰岛素 | |  | |  | | |  | |
|  | C-反应蛋白 | |  | |  | | |  | |
|  | 炎症因子 | |  | |  | | |  | |
|  | 白介素-2（IL-2） | | μg/L | |  | | |  | |
|  | 白介素-6（IL-6） | | μg/L | |  | | |  | |
|  | 肿瘤坏死因子（TNF-α） | | μg/L | |  | | |  | |
| **心电图(ECG)** | 心率 | QRS宽 | | PR间期 | | QT间期 | | | 总印象 |
|  | 次/分 | ms | | ms | | ms | | |  |

注：ECG总印象编号：0=正常 1=异常但无临床意义

2=异常有临床意义（请具体注明： ）
