# Supplementary material for: Obesity, cytokines and psychopathology in patients with chronic schizophrenia
Source: Front Psychiatry. 2025 Jul 28;16:1574041. doi: 10.3389/fpsyt.2025.1574041 (PMC12336245; doi:10.3389/fpsyt.2025.1574041)
Supplement: Supplementary file 2 [file Table2.docx]

**知情同意书**

**尊敬的病友及家属**：您好！

这是对患者治疗和康复非常有意义的项目，请您花数分钟时间阅读以下内容，依据您的想法做出决定，希望能得到您的理解和支持！

**1. 项目的意义：**

精神分裂症是一种严重影响患者的社会功能及日常生活的疾病，由于病因未明，目前只能对症治疗，疗效尚不够满意。认知损害严重影响患者精神康复和回归社会。本项研究通过测评患者的精神状况、认知状况、血液生化指标进行全面观察，以确认精神分裂症认知损害的风险因素，项目实施不会影响您的治疗方案，完全属于自然状态下的观察。

**2. 项目过程：**

对入组患者进行全面身体、精神检查，包括一系列的量表评定、精神状况评估、生化指标的测定。此过程需患者抽取静脉血10ml，用以测定项目涉及的血液生化和免疫指标。

**3. 危险性：**

抽取静脉血10ml，对患者基本无不良影响。

**4. 益处：**

整个项目实施的过程中的量表评定和血液生化免疫指标的测定均免费进行；而且可为患者及家属免费提供相关咨询。检测结果有利于医生对病人病情判断、调节药物剂量等。

**5. 自愿参加和拒绝：**

您的参加系自愿性质，您有拒绝参加和在任何时间退出本治疗的权力。如果您拒绝或退出，依然能够得到免费的相关的咨询服务，不会影响您在我院的治疗与康复。

**6. 保密性：**

我们会对您的资料进行保密，您个人相关临床资料及签署的知情同意书将由本项目的主持单位慎重保管。个人资料汇总分析后的结果将会在会议或出版物上报导，但我们会确保有关您的个人资料不会被公布。

**7. 联系人：**

如果您对本项项目有任何疑问，您可以与该患者所在科室主管大夫▁▁▁▁▁▁联系。

**8. 同意签字：**

如果您在下面签字或按手印，就表示您阅读了或请别人向您宣读了此知情同意书，理解了本项研究的目的和程序，您有机会对本项研究提出问题，并且表示您同意参加。

**签名或手印**： **日期：**____ 年 月 日

我向参与此项目的患者和/或其家属解释了知情同意书的内容，并认为研究对象理解了本项研究的目的、程序以及可能的危险和益处。

**研究人员签字**： **日期：**____年 月 日
